# Supplementary material for: What are we learning with Yoga? Mapping the scientific literature on Yoga using a vector-text-mining approach
Source: PLoS One. 2025 May 29;20(5):e0322791. doi: 10.1371/journal.pone.0322791 (PMC12121831; doi:10.1371/journal.pone.0322791)
Supplement: Attachment — Submitted filename: Rebutal_2025.pdf [file pone.0322791.s003.pdf]

Curitiba, February 17, 2025

Dear Kallol Kumar Bhattacharyya, MBBS MA PhD
Academic Editor of PLOS ONE,

We want to thank the reviewers for their excellent revisions, which have helped us to improve the manuscript in a general and definitive way. The feedback allowed us to improve its clarity and make it more informative for the community. We are sincerely grateful.

We are resubmitting the manuscript "**What We are Learning with Yoga: A Text Mining Approach to Literature,"** with the title changed to "**What are we learning with Yoga? Mapping the scientific literature on Yoga using a vector-text-mining approach**", as requested by reviewer 2. We have considered all suggestions and incorporated the necessary revisions into the manuscript. Below, we provide detailed responses to each comment, and all modifications have been highlighted in yellow in the manuscript for clarity.

We have organized the supplementary material and addressed the editor's requests to avoid any delays in a potential publication. We point out that to make it easier to access the study's supplementary material, we migrated our supplementary files (S1 to S7 and the code developed to construct the HTML-TMs) to the Zenodo platform, with DOI: 10.5281/zenodo.14763946 (https://zenodo.org/uploads/14763946), instead of the file "supplemental_files.zip" (see lines 330-332; 943-944). We have also added the code developed and used to obtain our results (S11_Codes.zip) as supplementary material at the same link, as requested.

Reviewer #1:

Thank you for the opportunity to review this interesting study and its methodology. I found the approach to be engaging and well-conceived. However, I would like to provide a few suggestions to enhance clarity and readability:

Abstract: The conclusion in the abstract appears somewhat vague. I would encourage you to provide a more precise and actionable takeaway to better communicate the key findings of the study.

Response: We have emphasized our main conclusion in the text of the abstract, which we believe reflects the most important findings of our study (lines 38-42).

Headings: In the "Background" section, I recommend using a more descriptive heading that directly reflects the content of the section. This will help guide readers more effectively through the material.

Response: We changed the section's title from 'Background' to 'Definitions and Technical Details' (line 105) to make it more transparent.

Results Section: To improve clarity, please consider structuring the results section more explicitly. Rather than referring to subgroups and groups using letters, it would be helpful to use descriptive names (e.g., "Respiration Group (A)"). This will make it easier for readers to follow and interpret your findings.

Response: To make the text easier to read and follow, we have added the full names of each group and sub-group throughout the text, along with the corresponding Roman numerals and letters.

Tables and Formatting: I recommend reviewing the APA guidelines for formatting tables and for italicizing statistical abbreviations. Ensuring consistency and adherence to APA standards will improve the overall presentation of your work.

Response: As requested, we changed all the tables to APA format and presented them in a standardized way.

Clearer Presentation of Groups: A clearer and more detailed presentation of the groups, including their definitions and distinctions, will significantly enhance the

reader's ability to engage with and understand your findings. I would e.g. appreciate some figure to visualize all groups.

I hope these suggestions are helpful as you refine your manuscript. Thank you again for sharing your research.

Response: We have restructured Table 1, which originally listed the dimensions and groups, to include subgroups (A, B, C, and D) and briefly describe each group. Further, we have incorporated the number of hits, unique occurrences, and coverage in Table 1, making the overlap between the defined groups more explicit. These additions improve clarity and help readers better understand the context of the yoga literature. Additionally, we have created Figure 4 to more effectively illustrate the overlap between the groups (lines 404–405, 411–41). Figure 3 also provides an overview of the groups, distinguished by color and their respective logical expressions.

Reviewer #2:

First and foremost, I would like to appreciate the authors' positive attitude towards the review process and constant efforts in revising and giving the manuscript a better shape. Their thorough attentiveness in analysing the descriptions and modification in the manuscript seems to be commendable. Their commitment for improving each aspect of the work from little details to extensive formatting seems praiseworthy. Now the manuscript provides a clearer and thorough vision. It can be said that the authors tried to provide an improved quality of the manuscript. However, there are some observations that needs attention-

• The manuscript explores research trends in Yoga, where the methodological approach for literature mining is majorly highlighted to develop a comprehensive framework for existing literature on Yoga, facilitating multidisciplinary collaboration and bringing new light to relevant aspects. Thus, the title of the manuscript need to be re-framed indicating the methodology (literature mining approach) for describing Yoga.

Response: We changed the manuscript's title from "**What We are Learning with Yoga: A Text Mining Approach to Literature**" to "**What are we learning with Yoga?**

Mapping the scientific literature on Yoga using a vector-text-mining approach", emphasizing our methodological approach.

• Define abbreviation e.g. BRET upon first appearance in the text.

Response: We have revised the acronyms in the document and corrected the passages where they were not defined when they first appeared.

• Authors have provided sub-sections like- Introduction and Background separately that needs to be improvised and re-organized following the IMRAD format.

Response: To incorporate the suggested IMRAD format, we have changed the 'Background' section to an introductory subsection, which we renamed 'Definitions and Technical Details' for clarity.

• The basic queries of the manuscripts are to understand Yoga's scientific foundation; the effects of Yoga practice be generalized to other fields, particularly health; identify underexplored topics within Yoga practice etc. However, it is difficult to get the direct corroboration with specific citations of the text mining results.

Response: To make it clearer what each stage of text mining contributes to obtaining our findings, we have drawn up Table S12 (placed as a supplement to the manuscript and deposited it on the Zenodo platform – DOI: 10.5281/zenodo.14763946). In this table, we clarify that the TM was a collaboration between the information presented by AI and human analysis, which helped refine the other. We modified our manuscript in lines 355-358 to present this addition. We would also point out that throughout the text, we have made other minor changes (highlighted in yellow) to emphasize the interpretation of the TM.

• Strengths and limitations of the study will help in improving the manuscript.

Response: Among the study's strengths, we emphasize that it provides a facilitator for the yoga researcher. We present an overview of Yoga for the first time.

UFPR
UNIVERSIDADE FEDERAL DO PARANÁ

Federal University of Parana
Graduate Program in Bioinformatics
Laboratory of Artificial Intelligence
Applied to Bioinformatics

Programa de Pós-graduação em
Bioinformática
UNIVERSIDADE FEDERAL DO PARANÁ

Our limitations relate to the exclusive use of titles and abstracts of studies on PubMed, as considerable literature may exist outside the platform. We did not carry out meta-analyses or deepen the analysis of the groups. Furthermore, our strategy may have omitted relevant areas, considering that we constructed the logical expressions (LOGEXP) from the survey of bibliographic reviews. Considering this, we have the prospect of using full articles in the mining, which would provide a deeper understanding of the field. We also aim to expand the technique, use the knowledge acquired in other areas of study, and offer the technique as a platform for users. In the discussion, we added a paragraph to clearly present the study's strengths, limitations, and perspectives. These are presented in lines 919-928.

We hope that the adjustments made, as suggested by the reviewers, have improved the manuscript's presentation and comprehensibility. Once again, thank the reviewers for their valuable feedback and contributions to help us improve the manuscript.

Kind regards,

Roberto Tadeu Raittz, Ph.D, Corresponding author
