# Supplementary material for: What are we learning with Yoga? Mapping the scientific literature on Yoga using a vector-text-mining approach
Source: PLoS One. 2025 May 29;20(5):e0322791. doi: 10.1371/journal.pone.0322791 (PMC12121831; doi:10.1371/journal.pone.0322791)
Supplement: Attachment — Submitted filename: Response.pdf [file pone.0322791.s004.pdf]

Federal University of Parana
Graduate Program in Bioinformatics
Laboratory of Artificial Intelligence
Applied to Bioinformatics

Curitiba, March 24, 2025

Dear Kallol Kumar Bhattacharyya, MBBS MA PhD
Academic Editor of PLOS ONE,

We are resubmitting the manuscript "**What are we learning with Yoga? Mapping the scientific literature on Yoga using a vector-text-mining approach**" to respond to the additional requests made by reviewer 1. We have considered all the suggestions and incorporated the necessary revisions into the manuscript, highlighting them in yellow. Below, we provide detailed responses to each comment.

We thank the reviewers and editors. The changes have greatly improved the document during this revision process.

Reviewer #1:

Thank you for your work on this paper. I found it very interesting to read, and it is clear that a great deal of effort has gone into this research. Although I am not an expert on the methodology used, it appears to be sound and rigorous, making the paper a strong candidate for publication. I do, however, have a few minor suggestions that could improve clarity and readability:

We thank the reviewer for his suggestions and hope that we have met his expectations.

Tables and Abbreviations
When using titles in tables, figures, or similar elements, please ensure that all abbreviations are explicitly defined. For example:
Table 2. Mapped Relations Between YP and Positive Neurobiological Effects (YP = Yoga Practice).
Response: We have incorporated the full meaning of the acronyms into the figures' and tables' titles and captions. Modifications are highlighted in yellow (see lines 242-3,377,412,416,562,780-1).

Effect Sizes
I would appreciate it if effect sizes were included wherever available. If they are not reported, please mention this explicitly to provide full transparency.
Response: We appreciate the reviewer's suggestion about including effect sizes. However, it is important to note that the methodology proposed in this study does not involve hypothesis testing or association analyses since our focus was on text mining. The main objective was to contextualize and demonstrate the relevance of the articles in the field of yoga without going into statistical depth. As such, effect size analysis does not apply to the scope of this work. We suggest that readers consult the original articles for more detailed statistical information. To ensure greater clarity, we emphasized that our review prioritized a qualitative approach through text mining (lines 924,928). We hope this explanation clarifies our methodological approach.

Table Formatting and Ordering
Table 3 does not follow APA style. Please revise it accordingly.
The order of elements in Table 3 and Appendix B seems somewhat arbitrary. Organizing them by te number of studies found, year of publication or alphabetically could improve readability. If a specific rationale for the current order exists, please clarify it.
Response: We changed the order of the elements in Table 3, respecting the alphabetical order. We also formatted Table 3 to conform to APA format, inserting two columns with the number of documents and titles, making the information more straightforward to read.
We checked Supplementary Table S12, and the order is in line with the sequence in which the study was carried out, which is made clear in the text (lines 358-9). However, we could not identify what the comment about Appendix B refers to.

Discussion Section Clarity
Some parts of the discussion are difficult to follow. For instance, the first sentence:
"Exploring topics related to yoga through vector representations and semantic investigations reveals that yoga has a scientific foundation, addressing the first question."
The phrase "the first question" is unclear. Readers should not have to search through the document to understand what is being referenced. Please consider rewording for greater clarity.
Response: We appreciate the reviewer's suggestion, and for clarity, we have left questions 1 and 2, mentioned in the discussion, explicit (lines 787-8,837-8).

Headings in the Discussion Section
Adding subheadings, such as Strengths & Weaknesses, would help structure the discussion and make it easier to navigate.
Response: We have subdivided the discussion into three topics to make it easier to navigate the text. The subsections were 4.1 Organizing Yoga Research (line 805), 4.2 Underexplored Aspects of Yoga Practice (line 873), and 4.3 Strengths, Challenges, and Future Directions (line 925).

Best regards,

Roberto Tadeu Raittz, PhD corresponding author
