# Supplementary material for: What are we learning with Yoga? Mapping the scientific literature on Yoga using a vector-text-mining approach
Source: PLoS One. 2025 May 29;20(5):e0322791. doi: 10.1371/journal.pone.0322791 (PMC12121831; doi:10.1371/journal.pone.0322791)
Supplement: Attachment — Submitted filename: Reviewers_answer.docx [file pone.0322791.s001.docx]

Federal University of Paraná

**Dear Editor Dr. Hilary Genn Givero,**

We are sending the rebuttal letter as the response to the reviewers of the manuscript entitled "**What we are learning with Yoga: A text mining approach to Literature**". The manuscript was previously submitted under ID PONE-D-24-47636 and is currently under ID PONE-D-24-10403 in a new submission.

We want to thank the reviewers for their valuable suggestions, highlighting essential points in the manuscript that needed more clarity, leading to an overall improvement. The manuscript has undergone significant revision in structure and sentences to improve clarity. We have revised several sections to improve the overall flow and readability of the text. In addition, the HTML from text mining initially provided as part of the supplementary material is now accessible on a user-friendly web platform at <https://aibialab.github.io/HTMLTM_Yoga>.

We have responded to the reviewers and made the changes they suggested. We have added two new topics (sections 2 and 4.3) and highlighted them in yellow. Section 2, Background, aims to make our methodology easier to understand by describing the main techniques used (lines 98-204). Section 4.3, Yoga Styles and the focus of the literature, in response to the request of the reviewers, describes the main yoga styles and what the literature addresses about each of them (lines 745-756).

Additionally, we changed our manuscript title from "What we are learning with Yoga: A text mining approach to Literature" to "**What are we learning with Yoga? A text mining approach to Literature**".

Below are the reviewers' comments and our responses and considerations.

**REVIEWER 1**

Reviewer #1: The authors took a nice initiative in exploring YP data mining, as this area needs to be explored in the present scenario. Related authentic information needs to be displayed on the board so that academicians, researchers, and planners may take the initiative to plan, develop, and carry out studies in the field with more focus. Yoga, an ancient practice originating from India, has seen a global surge in popularity over the past few decades. It encompasses various rules, and regulations, along with Asanas (postures) and Mudras (poses) like physical postures (asanas), breath control and breathing styles (inhale and exhale pranayama), and meditation (dhyana). The aim is to harmonize the body, mind, and spirit. And, can be easily adopted in any culture. The widespread adoption of yoga across different cultures and societies worldwide has been highlighted; and, such manuscripts may help the scientific community to identify and acknowledge the gap to plan further with concerted efforts. However, I have some suggestions that may help in improving the content and attracting the learned academicians and scientists of the field.

Response: Dear reviewer, We thank you for your positive comments and helpful suggestions. They have been instrumental in enhancing the clarity and depth of the study. We restructured the manuscript to improve clarity and highlight the scope of the study.

We have made significant improvements to the manuscript, particularly in the Introduction and Methodology sections, and have clarified the focus of the study. We have included a section called "Background" to make the methodology more comprehensible to readers unfamiliar with some aspects of the method. Furthermore, we have enhanced the cohesion and contextualization of the literature in the Results and Discussion sections and included section 4.3 on the approach to yoga styles in the literature.

The manuscript needs thorough scanning as some places sentences seem incomplete; hence, it would be appropriate to read the entire article carefully and check the syntax. Also, at many places to provide evidence, instead of the text authors used reference no. which seems inappropriate in a scientific manuscript.

Response: Thank you for your comments. We have comprehensively reviewed the manuscript to address the issues raised. We have revised several sentences to improve clarity and enhance the text's overall structure, ensuring a more polished use of English and the correct format of references. For instance, in the mentioned example, we changed:

From: “However, our analysis found elements in Embodied cognition theory that can corroborate the elaboration of new frameworks and strategies to design studies about Yoga. The analysis of social contexts in which practitioners may be included is addressed by [86] and [85]; the relevance of the benefits that self-care based on yoga techniques offers to accessibility is discussed by [87]. Yoga in work settings and interpersonal relationships is contextualized by [88] and [89]. We also found papers addressing ethical aspects present in traditional principles of yoga, like in [90] study”

To: "However, our analysis found elements of embodied cognition theory that can support elaborating new frameworks and strategies for designing studies on Yoga. These studies range from analyzing the social contexts of practitioners [94,95] to the relevance of the benefits of self-care based on yoga techniques to accessibility [96], including yoga in work settings and interpersonal relationships [97,98], and the ethical aspects present in the traditional principles of yoga [99]."

**REVIEWER 2**

Reviewer #2: The paper appears to have conducted a thorough analysis of the existing literature on yoga in which the study questions have been answered. Due to my poor understanding of the methodology used, it is difficult for me to assess its correct use. However, I would like to point out that the paper would benefit from indicating in the methodology section at the outset what kind of study they are going to carry out and exactly which part of the articles they are going to analyse. In addition, they could indicate the number of articles excluded. On the other hand, the authors attach all the necessary information with which they have carried out the research.

Response: Dear reviewer, We thank you for your valuable comments and suggestions.

We have included a diagram (Figure 2) to clarify our methodology and enhance its understanding. Additionally, we have expanded Section 2, titled "Background," to provide a more comprehensive overview of the tools utilized in our methodology, mainly focusing on the Biotext and SWeeP tools (lines 98-140). We indicated the sizes of the sets used in the text (lines 210-221 and in Figure 2). Our initial search in PubMed was for articles containing "yoga" in the title or abstract, which returned 6,905 documents used in a preliminary phase of our research. We then filtered this to include only those with "yoga" in the title, ensuring the study's focus remained on yoga. This new set resulted in 2,929 documents, which formed the basis for most of our analyses. As we created a platform for accessing the yoga literature, we should include all these papers as subjects for the TM analysis. We hope these changes have made the text clearer.

Regarding the wording, it is correct but I have detected the following errors: line 133 correct the word tree and lines 426-429 seem to form the same sentence

Response: Thank you for bringing these mistakes to our attention. We have made the necessary corrections.

The results are well developed and answer the study questions. In this section four dimensions are proposed, which the authors use to categorise the existing literature.

Response: Thank you for your positive comments.

Regarding the discussion: no thorough comparison with existing literature has been made (if there is none because the methodology is very new, this should also be indicated). It should be analysed whether the questions could have been answered well with the chosen method and whether any errors could have been made in the process. Indicate possible limitations in conducting the study. In my opinion it seems to be a continuation of the results, rather than a discussion of them, so I suggest that the authors revise this section and better substantiate their results by comparing them with other studies.

Response: We thank the reviewer. The suggestions on these subjects were crucial to the improvement of our manuscript. The methodology used in this study is innovative since it is the first formal application of this method in the field. Therefore, there is a lack of direct comparative studies in the literature.

We want to emphasize that we have made significant changes to the manuscript so that we believe these issues were addressed and resolved during rewriting. Therefore, we have restructured the manuscript to improve the clarity of the content, highlight the study's scope, and relate the results to the discussion.

The indicated limitations of the study are now addressed in the text. About the lack of evaluation of the quality of articles, we justify it because it was outside the scope of this study once we should cover all literature. Our main objective is to contextualize the yoga literature in its subtopics instead of evaluating the specific quality of the selected articles and their contribution to each subtopic of the yoga field (see lines 765-768). The other limitation is the lack of studies systematically evaluating yoga's preventive benefits and positive effects on joint health, such as mobility and flexibility (see lines 842-851).

Overall, our study intends to contribute to understanding the current scientific literature on yoga. The text mining and vector representation methodology has proven to be a powerful tool for analyzing large amounts of data and identifying semantic relationships between key concepts. The developed HTML-TM platforms will provide a valuable resource for researchers in the field, facilitating navigation, contextualization, and in-depth analysis of the literature on yoga.

**REVIEWER 3**

Reviewer #3: The reviewer greatly appreciates the amount of time and effort the authors put into preparing the manuscript and agree that the study of yoga would benefit from more multidisciplinary collaboration.

Response: Dear reviewer, thank you for your kind comments. We aimed for this objective when we conceived the study.

However, while the text-mining method is an interesting approach to examining the breadth of the yoga literature, the manuscript itself was challenging to understand and would benefit from significant revisions to improve readability. The authors' interpretation of their findings was challenging to evaluate due to significant usage of vague and ambiguous language, leaving the reader confused as to what interpretations or analyses were being made.

Response: Thank you for your comments. We have conducted a comprehensive and meticulous revision of the manuscript. We have revised several passages to enhance clarity and improve our use of English, ensuring that the text is well structured.

Furthermore, the authors have crucially missed any evaluation of the quality of the literature reviewed. Given the vast breadth of poor-quality yoga research, as noted by the authors as well, the yoga research community would benefit from limiting the number of articles that re-iterate the findings of a poorly designed trials, without discussing the quality of those findings.

Response: Thank you for your comments. Regarding the quality of the articles under review, despite all articles being obtained from the PubMed platform from indexed journals, we concur with the reviewer that some articles are of lesser quality in the existing literature. Therefore, we have elected to include the full literature in our analysis because of the main reason we presented to reviewer 2 (see lines 765-768). As we created a platform for accessing the yoga literature, we should include all the papers in TextDoc as subjects for the TM analysis. So, we advised the readers to attempt to meet this limitation, which we consider beyond the scope of our study. We have also clarified this issue in our manuscript on lines 765-768.

Below are cited a few specific examples for the authors' consideration:
Major comments

It was unclear how the 3 fundamental research questions [pg. 2] were evaluated, specifically:
a. "Does yoga have a solid scientific foundation?" What do the authors mean by a 'scientific foundation?'. Based on the discussion section (lines 493-496), it appears the authors suggest that a scientific foundation is granted simply due to the existence of yoga research within literature. The reviewer suggests this analysis is harmful to the study of yoga as a science and suggests that the authors consider evaluating what specific criteria merit having a solid scientific foundation.

Response: We appreciate the reviewer's observation. When we say that yoga has a "solid scientific basis," we mean that it is a recognized object of study, as evidenced by the 6,905 articles retrieved in our initial research phase. The substantial body of research in the field allows us to assert yoga's scientific relevance confidently. In our study, we found several studies investigating the effects of yoga on mental health, physical well-being, stress management, and other health conditions. We view yoga as an object of study that encompasses research from controlled clinical trials to systematic reviews, and many of these studies indicate significant benefits associated with yoga practice. Furthermore, studies conducted by various research teams that have yielded comparable outcomes with yoga practice (YP) reinforce its scientific basis. However, efforts to link yoga to multidisciplinary aspects are still lacking.

The quality and methodology of the studies can vary, and it is crucial to consider the evidence within the context of each study. We acknowledge that yoga is a practice with a high level of popularity, which has led to its adaptation in various forms that may, sometimes, deviate from the original practice and philosophy. Therefore, the diversity of contexts and applications of yoga globally does not negate its practice's potential benefits and its study's importance. We emphasize this in the discussion on lines 759 to 768.

2. While this type of analysis does not require an evaluation of the quality of the articles included, the yoga research community would benefit from limiting the number of studies that re-iterate the findings of a poorly designed trials, without acknowledging the quality of those findings. While the reviewer can appreciate the benefit of mapping semantic relationships between yoga findings, this manuscript reports the common relationships that appear in the literature, but not the quality of those relationships which may lead future researchers in the wrong direction. Below is an example of simply listing findings with no discussion of the quality of these findings, evaluation of the relationships or the application of these findings to the research questions.

a. P. 9 "The concepts of self-image and embodiment stand out in theoretical studies about yoga in psychology. Some examples are papers concerning obesity [7, 10, 84, 85]. However, our analysis found elements in Embodied cognition theory that can corroborate the elaboration of new frameworks and strategies to design studies about Yoga. The analysis of social contexts in which practitioners may be included is addressed by [86] and [85]; the relevance of the benefits that self-care based on yoga techniques offers to accessibility is discussed by [87]. Yoga in work settings and interpersonal relationships is contextualized by [88] and [89]. We also found papers addressing ethical aspects present in traditional principles of yoga, like in [90] study"

Response: We appreciate your input on this crucial matter. We consider it vital for future researchers to assess the quality of articles, as poor-quality studies can lead to erroneous conclusions. To mitigate the influence of individual statements in each article, we have revised our manuscript to prioritize contextually supported results. In this way, we aimed to reduce the impact of specific findings while focusing on the overall pattern of aspects involved. This effort included the paragraph above highlighted in lines 576-584. So, the article quality assessment was not within this project's scope mainly because of the needs of the text mining defined scope that aims to describe the literature as it is. For this, many studies were cited as examples in the literature rather than conclusive by themselves.

3. The manuscript included multiple instances of vague/ambiguous phrasing making it hard for the reader to understand the analysis or interpretation the author was trying to convey. A few examples below:
a. P. 7 "The LOGEXP-77 ('HEMODYNAM') brings only 18 documents. Still, it shows the Data Mining Model's capacity to infer unexplicit relations, as in [34]" The authors don't specify what is being shown, what relationships are being inferred or include any specific examples from the reference [34] cited, leaving the reader guessing and hunting through that reference to attempt to make the inference.

Response: Thank you for pointing out these issues. We addressed them, the manuscript has been comprehensively revised, and the ambiguous sentences, including the example previously cited, have been corrected. Please refer to the revised version of this example on lines 501-505.

b. P. 10 "Furthermore, many of these markers have already been presented, reinforcing the advanced possibility that YP-related characteristics also correlate analogously. If we abide by this principle, we can trace the possible relationships by traversing the evidence provided by the markers on the various research fronts, inferring new potential relationships. These efforts are beyond this paper's scope, but we think it is a promising avenue for further studies" There is no discussion here of which YP-related characteristics, what the relationship are or what inferences are being made.

Response: We thank the reviewer for raising this point, which calls for improving clarity. We have revised the text (lines 612-618) to simplify our intent. Our aim with these statements was to emphasize that we provided a tool for future yoga researchers to make inferences in the literature beyond what we have studied and presented in the paper. The HTML-TM was initially available as part of the supplementary material, and now the resource is accessible on a user-friendly web platform at <https://aibialab.github.io/HTMLTM_Yoga>. We believe this tool will facilitate future yoga research by identifying new relationships and gaps in the literature that warrant further study. We hope we have made this point clear.

4. The reviewer greatly appreciates that "This study aims to elaborate a global structure of the literature on Yoga to open the door for multidisciplinary collaboration and bring new light to some relevant aspects." [pg. 1] and agrees that the study of yoga would benefit from more multidisciplinary collaboration.

Response: Thank you for the positive comments.

However, a significant limitation to the approach utilized was the examination of yoga as a single entity or practice, when the practice of yoga varies widely across traditions, styles, and branches of practice (see Gard et al., 2014 for an example of how health outcomes might be considered within the context of stylistic differences). While reviews of the yoga literature do generally require a reduction in search terms to "yoga", this study was significantly lacking in accounting for the significant variety of yoga approaches (e.g. hatha yoga, Iyengar yoga, Ashtanga yoga, Kriya yoga to name just a few) and the vast differences that these approaches can have on health-related outcomes. The analysis of the four primary dimensions would have benefitted significantly from additional exploration of the stylistic differences of the studies included within those dimensions.

Response: We appreciate the comment and recognize the relevance of the point raised about various yoga styles and their different approaches. To better address this point, we have added section 4.3, which examines how the literature approaches different styles of yoga. However, most studies treat yoga generically without clearly distinguishing between specific styles. To clarify this, we have included Table 3, which shows the main focus of the literature for each style of yoga and counts the number of studies per style. We found that, in general, studies focus on Hatha yoga (including hot yoga) and Iyengar yoga. At the same time, the other styles are poorly represented in the literature.

5. The figures, particularly the smaller text, were blurry and almost impossible to read.

Response: Thank you for noticing the need to improve the images. We are now supplying vector images with adequate resolution.

With our best regards,

RobertoTadeu Raittz

(Corresponding author)
