# Supplementary material for: What are we learning with Yoga? Mapping the scientific literature on Yoga using a vector-text-mining approach
Source: PLoS One. 2025 May 29;20(5):e0322791. doi: 10.1371/journal.pone.0322791 (PMC12121831; doi:10.1371/journal.pone.0322791)
Supplement: Attachment — Submitted filename: PONE review_14-01-2024.docx [file pone.0322791.s002.docx]

First and foremost, I would like to appreciate the authors’ positive attitude towards the review process and constant efforts in revising and giving the manuscript a better shape. Their thorough attentiveness in analysing the descriptions and modification in the manuscript seems to be commendable. Their commitment for improving each aspect of the work from little details to extensive formatting seems praiseworthy. Now the manuscript provides a clearer and thorough vision. It can be said that the authors tried to provide an improved quality of the manuscript. However, there are some observations that needs attention-

- The manuscript explores research trends in Yoga, where the methodological approach for literature mining is majorly highlighted to develop a comprehensive framework for existing literature on yoga, facilitating multidisciplinary collaboration and bringing new light to relevant aspects. Thus, the title of the manuscript need to be re-framed indicating the methodology (literature mining approach) for describing Yoga.
- Define abbreviation e.g. BRET upon first appearance in the text.
- Authors have provided sub-sections like- Introduction and Background separately that needs to be improvised and re-organized following the IMRAD format.
- The basic queries of the manuscripts are to understand Yoga’s scientific foundation; the effects of Yoga practice be generalized to other fields, particularly health; identify underexplored topics within Yoga practice etc. However, it is difficult to get the direct corroboration with specific citations of the text mining results.
- Strengths and limitations of the study will help in improving the manuscript.
